# Feature selection for helpfulness prediction of online product reviews: An empirical study

**DOI:** 10.1371/journal.pone.0226902

**Published:** 2019-12-23

**Authors:** Jiahua Du, Jia Rong, Sandra Michalska, Hua Wang, Yanchun Zhang

**Affiliations:** 1 Institute of Sustainable Industries & Liveable Cities, Victoria University, Melbourne, VIC, Australia; 2 Faculty of Information Technology, Monash University, Clayton, VIC, Australia; College of EME, NUST, PAKISTAN

## Abstract

Online product reviews underpin nearly all e-shopping activities. The high volume of data, as well as various online review quality, puts growing pressure on automated approaches for informative content prioritization. Despite a substantial body of literature on review helpfulness prediction, the rationale behind specific feature selection is largely under-studied. Also, the current works tend to concentrate on domain- and/or platform-*dependent* feature curation, lacking wider generalization. Moreover, the issue of result comparability and reproducibility occurs due to frequent data and source code unavailability. This study addresses the gaps through the most comprehensive feature identification, evaluation, and selection. To this end, the 30 most frequently used content-based features are first identified from 149 relevant research papers and grouped into five coherent categories. The features are then selected to perform helpfulness prediction on six domains of the largest publicly available Amazon 5-core dataset. Three scenarios for feature selection are considered: (i) individual features, (ii) features within each category, and (iii) all features. Empirical results demonstrate that semantics plays a dominant role in predicting informative reviews, followed by sentiment, and other features. Finally, feature combination patterns and selection guidelines across domains are summarized to enhance customer experience in today’s prevalent e-commerce environment. The computational framework for helpfulness prediction used in the study have been released to facilitate result comparability and reproducibility.

## Introduction

Customer product reviews play a significant role in today’s e-commerce world, greatly assisting in online shopping activities. According to a survey conducted in 2016 [1], 91% of online shoppers read product reviews while searching for goods and services, and 84% of them believe that the reviews are equally trustworthy as recommendations from their friends. Online reviews do not only enhance the customer purchasing experience through valuable feedback provision, but also facilitate future product development activities by better understanding the customer needs.

Online product reviews are also highly susceptible to quality control [2], which can potentially harm online shopping experience. A recent study [3] shows that users tend to limit their attention to only first few reviews, regardless of their helpfulness. It is generally viewed that helpful reviews have more impact on customers’ final decisions. However, the large and overwhelming nature of online product reviews makes it difficult for customers to efficiently locate useful information. Although the majority of online platforms enable review helpfulness assessment through user voting, the large proportion of records does not contain any votes. The scarcity of user votes is even more noticeable for less popular products.

Automatic helpfulness prediction helps consumers identify high-quality reviews, which has attracted substantial attention. The mainstream approach follows a procedure of careful feature curation from multiple data sources [4]. Still, the features are frequently domain- and/or platform-dependent, substantially inhibiting wider application. Also, the features are selected arbitrarily without solid justification. Furthermore, prior research mainly focuses on the predictive power of the entire feature set, while little is known on the contribution and necessity of using individual or subsets of features. Since identical feature set is rarely used among existing studies, the reported results prove challenging for fair comparison. Finally, the existing studies are often conducted on publicly unavailable ad-hoc datasets, hampering result reproducibility.

To address the aforementioned gaps, this study comprehensively identifies, evaluates, and selects representative features for helpfulness prediction. Specifically, frequently used domain- and platform-*independent* features (i.e., content-based features) are first identified from considerable recent literature. The predictive power of the identified features is then evaluated on six domains of large-scale online product reviews. Instead of evaluating the entire feature set, the study allows for performance-oriented feature selection under multiple scenarios. Such flexibility can effectively justify (not) selecting certain features. As a result, feature combination patterns and selection guidelines across domains are summarized, offering valuable insights into general feature selection for helpfulness prediction. The publicly available source code and datasets ensure result comparability and reproducibility of the study.

This study contributes to existing literature in four aspects:

- First, the study conducts one of the most comprehensive literature reviews on helpfulness analysis to identify frequently used content-based features.
- Second, the study conducts the first and most extensive empirical validation on large-scale publicly available online product reviews to report feature behaviors in multiple scenarios (individual and combinations) and domains.
- Third, a holistic computational framework is developed for helpfulness prediction from scratch, including data pre-processing, extracting the identified features, and evaluating the predictive power of individual features and feature combinations.
- Fourth, the source code, dataset splits, pre-processed reviews, and extracted features have been released for result reproducibility, benchmark studies, and further improvement.

The remaining of the study is organized as follows. The Related work section surveys recent literature regarding the use of features and feature selection for review helpfulness prediction. The Methodology section introduces steps for approaching feature-based helpfulness prediction, including feature identification, feature extraction, and feature selection strategies used in the study. Substantial analysis is conducted in the Empirical analysis section. Empirical results are reported and discussed to evaluate and locate optimal feature combinations, followed by frequent pattern discovery. Subsequently, the study summarizes the implications and discusses the limitations in the Implications and Limitations section, respectively. Finally, the Conclusions and future works section encapsulates the findings and outlines future directions of the study.

## Related work

The automatic prediction of reviews helpfulness is majorly approached via feature engineering. Previous studies have curated a large body of features derived from (i) review content [5–10] and (ii) review contexts such as reviewers [11, 12], social networks among reviewers [13, 14], review metadata [15, 16], and product metadata [17, 18]. Some other less frequent contextual features include review photos [19, 20], manager responses [21], travel distances [22], to name a few. This study focuses on content-based features due to the ubiquitous use in literature and the ability of review texts to generalize across online platforms.

Recent studies regarding helpfulness prediction and feature selection have been identified and summarized. Kim et al. [5] investigated the effect of ten features spanning four main categories (i.e., lexical, structural, semantic, syntactic and metadata), and their combinations on helpfulness prediction. The authors found out that the most useful features were review length, unigrams, and product ratings. Zeng et al. [23] reported the results of individual features and all-minus-one feature combinations. They introduced “the degree of detail” feature as a function of review length and *n*-grams, alongside seven other features. The introduced feature proved to be the most important in helpfulness prediction, leading to a significant drop in accuracy after its exclusion. Yang et al. [8] evaluated the impact of review structure, unigrams, and three sentiment features: Geneva Affect Label Coder, Linguistic Inquiry and Word Count, and General Inquirer. The latter two features not only improved the prediction performance, but also provided a useful interpretation to what makes a review helpful.

Akbarabadi et al. [24] focused on 12 features from the review characteristics category, including review length, review age, part-of-speech, richness, sentiment and readability. The title characteristics category was also introduced, which did not improve the performance of helpfulness prediction. Vo et al. [25] investigated the four feature categories, namely anatomical, metadata, lexical and added feature group, which included (i) the number of helpfulness votes, and (ii) the number of positive and negative words. The impact of (i) on prediction accuracy proved to depend on both datasets and the choice of classifiers. The results for (ii) demonstrated a similar pattern.

Haque et al. [26] analyzed the performance of lexical, structural, semantic and readability feature groups. The last group was added in order to unfold the complexity of the review content, and showed significant impact on helpfulness prediction. Chen et al. [27] adopted the features related to text surface (i.e., the number of words, sentences, exclamation marks, question marks, and uppercase, lowercase), unigrams, part-of-speech, and word embeddings. The word embedding features trained using the Skip-gram model outperformed unigrams on an opinion spam detection dataset collected from amazon.

In terms of neural network-based models, Fan et al. [28] conducted helpful review identification based on recurrent neural networks, using the metadata of their target products. Saumya et al. [29] developed a two-layer convolutional model upon both the Skip-gram and Global Vectors model. Still, such approaches lack interpretability, making it difficult to identify what particular aspects of the reviews are good indicators of helpfulness.

As presented above, the numerous analysis tasks have been conducted to extract the most useful features for helpfulness prediction. However, the research within domain is often fragmented and heterogeneous, which challenges the objective comparison and findings synthetization. For example, the categorization of features differs among the studies, impacting finding generalizability. Also, the features selected in prior research frequently lacks justification behind particular feature selection, leading to the potential bias in results interpretation. Moreover, most of the existing studies suffer from result reproducibility due to the unavailability of ad-hoc datasets and implementation details.

Given the limitations identified, this study (1) provides the most comprehensive and generalizable content-based feature set evaluation on large-scale publicly available datasets, (2) conducts the empirical validation of the most effective feature selection in an objective manner, and (3) releases the datasets and source code describing the implementation details used in this study.

To the best of our knowledge, this study is the first to address the reproducibility and transferability issue of review helpfulness prediction, as well as the first work that provides the justification-driven feature selection process regardless of the platform and domain of applications. The complete and systematic literature review proves practically infeasible given largely fragmented state of the research in helpfulness prediction domain. Still, the study has made best efforts to report the latest state-of-art and identify the gaps to fill with the current work.

## Methodology

Feature-based helpfulness prediction entails three steps. To start with, the procedure and criteria are described to collect recent relevant literature, from which frequently cited content-based feature candidates are identified. Each of the identified feature candidates is then introduced and the feature construction process is specified. Finally, the evaluation protocols and feature selection strategies are provided to locate optimal feature combinations for review helpfulness prediction.

### Feature identification

The study identifies frequently cited feature candidates from recent literature to provide wide generalization and fair comparison with the majority of studies on the topic. To this end, a collection of most recent relevant studies are first collected and filtered, from which feature candidates are identified.

**Paper acquisition** The collection of relevant papers is based on (i) the references of the three most recent survey papers from the review helpfulness field [4, 30, 31] and (ii) the top 50 relevant studies retrieved from the Google Scholar database and published before 2019, using the following search query:
  (“*online reviews*” OR “*product reviews*” OR “*user review*” OR “*customer review*” OR “*consumer reviews*”) AND (“*useful*” OR “*helpful*” OR “*usefulness*” OR “*helpfulness*”).
  Given the scope of the study, the 149 collected papers are filtered based on the following criteria: (i) *automated* prediction of online product review helpfulness; (ii) inclusion of *factors* influencing review helpfulness; and (iii) *English-written* review analysis only. As a result, 74 papers (See the “Literature” column in Table 1) are identified.
**Feature acquisition** Features mentioned in the 74 identified papers are collected, along with the frequency of feature mentions. The following rules are adopted for feature list compilation: (i) features mentioned at least three times over the entire paper collection to exclude rare features, (ii) removal of human-annotated features due to expensive manual annotation process, and (iii) inclusion of only *content-based* features to support platform-independent generalizability and transferability. As a results, 27 feature candidates are identified.

**Table 1:** Features used in the analysis.

Category	Feature	Dimension	Description	Literature
Semantics	UGR	*V*	Unigram TF-IDF	[5, 7, 8, 13, 23, 26, 32–43, 88]
	BGR	*V*^2^	Bigram TF-IDF	[5, 23, 26, 40, 42, 44]
	LDA	100	LDA topic distribution	[37, 39, 45–48, 88]
	SGNS	300	Skip-gram Negative Sampling	—
	GV	300	Global Vectors	—
Sentiment	LIWC	93	Linguistic Inquiry and Word Count dictionary	[8, 35, 49–55, 88]
	GI	182	General Inquirer	[5, 8, 18, 33, 39, 42, 56, 88]
	GALC	21	Geneva Affect Label Coder	[7, 8, 33]
	OL	3	Opinion lexicon	[13, 38, 40, 57–59]
	SWN	3	SentiWordNet	[37, 59–62]
	SS	3	SentiStrength	[10, 63, 64]
	VADER	3	VADER lexicon	—
Readability	FKRE	1	Flesch–Kincaid Reading Ease score	[7, 10, 18, 29, 35, 38–40, 48, 51, 54, 59, 62, 65–67]
	FKGL	1	Flesch–Kincaid Grade level	[9, 10, 35, 38, 39, 65, 68]
	GFI	1	Gunning Fog Index	[9, 10, 16, 38, 40, 50, 52, 53, 65, 66, 68]
	SMOG	1	Simple Measure of Gobbledygook	[9, 10, 38–40, 65]
	ARI	1	Automated Readability Index	[9, 10, 38, 52, 56, 65, 66, 68, 69]
	CLI	1	Coleman–Liau Index	[9, 10, 15, 38, 49, 52, 65, 66, 68, 70]
Structure	CHAR	1	Number of characters	[26, 35, 45, 52, 65, 71–75]
	WORD	1	Number of words	[5, 7, 8, 11, 13, 15, 16, 18–21, 23, 26, 29, 32, 33, 35, 37–43, 45, 48–50, 52, 53, 56–58, 60, 62, 63, 65–71, 76–82, 88]
	SENT	1	Number of sentences	[5, 7, 8, 13, 21, 26, 32, 33, 35, 37, 39–43, 45, 52, 57, 58, 65, 71, 79, 88]
	AVG	1	Average number of words per sentence	[5, 8, 13, 33, 35, 39, 40, 43, 45, 52, 57, 58, 71, 79, 83, 88]
	EXCLAM	1	Number of exclamatory sentences	[5, 8, 33, 41, 42, 88]
	INTERRO	1	Number of interrogative sentences	[5, 8, 26, 33, 41, 42, 88]
	MIS	1	Number of misspelling words	[38, 39, 54, 59, 65, 71]
Syntax	NOUN	1	Number of nouns	[5, 7, 10, 13, 29, 32, 39, 42, 43, 52, 59]
	VERB	1	Number of verbs	[5, 7, 10, 13, 29, 32, 39, 41–43, 59, 84]
	ADJ	1	Number of adjectives	[5, 7, 10, 13, 29, 32, 39, 41–43, 58, 59]
	ADV	1	Number of adverbs	[5, 7, 10, 13, 32, 39, 41–43, 58]
	COMP	1	Number of comparative sentences	[13, 23, 32, 43, 55, 85]

*V* indicates the vocabulary size of the training set of a corpus.

As a novelty, the study additionally incorporates two semantic features and one sentiment feature that are gaining more recent attention. Such features have been proved robust in numerous text mining and natural language processing applications but are so far under-studied in review helpfulness prediction.

Table 1 presents the 30 content-based features identified from recent literature. The features are further grouped into five coherent categories (i.e., semantics, sentiment, readability, structure, and syntax) following the convention in the research field.

Note that context-based features such as reviewer characteristics are currently excluded from the feature pool since they are domain- and/or platform-dependent, and thus not always available.

### Feature extraction

The description and construction process of the identified features in groups is presented as follows. It is worth noting that some features overlap functionally, for instance, all sentiment features compute the emotional composition of reviews via different lexicons. Some features are constituents of others, such as readability scores resulting from different linear transformations of certain structural features. Following the convention in the research field, features in both cases are treated as individual ones.

#### Semantics

Semantic features refer to the meaning of words and topical concepts from the review content by modelling terms statistics into vectors. The five semantic features for the helpfulness prediction task are as follows:

**UGR and BGR** The unigram bag-of-words representation of a review uses the term frequency-inverse document frequency (TF-IDF) weighting scheme [86], where each element of a vector corresponds to a word in the vocabulary. Similarly, the bigram bag-of-words representation encodes all possible word pairs formed from neighboring words in a corpus. Both UGR and BGR ignore terms that have a document frequency value below 10 when building the vocabulary. The vector representations are then transformed into unit vectors via the L2 normalization.
**LDA** Latent Dirichlet Allocation representation learns the topic distribution of a review. Topic modeling considers corpus as a mixture of topics, and each topic consists of a set of words. In the case of online product reviews, the topics can be different product properties, emotional expressions, etc. The original LDA algorithm [87] is adopted to learn the probability distribution of latent topics for each review. Following [88], the number of topics is set to 100 during training.
**SGNS and GV** As a novelty, the study also uses the two most recent types of *word embeddings* as features. The Skip-Gram with Negative Sampling [89] and Global Vectors [90] aim at learning the distributed representations of words. Under this setting, each word is mapped into a dense vector space, where similar terms display closer spatial distance. Thus, each review can be simply converted into a vector by averaging the embeddings of its constituent words, where out-of-vocabulary words are skipped.

#### Sentiment

Sentiment features analyze the subjectivity, valence, and emotion status of content written by customers. Previous works [22, 91] have shown relevance between helpfulness of a review and the sentiments expressed through its words. The study constructs sentiment features using the seven most frequently-used lexicons. The first three lexicons are category-based, each estimating the probability of a review belonging to its predefined lexicon categories. The remaining lexicons are valence-based, each looking up the valence (i.e., positive, neutral, and negative) of words in a review where possible. Note that both the categories and word valence are defined differently among lexicons. As a result, the seven sentiment features will lead to different vector representations due to various measurement criteria.

**LIWC** The Linguistic Inquiry and Word Count dictionary [92] classifies contemporary English words into 93 categories, including social and psychological states. The dictionary covers almost 6, 400 words, word stems, and selected emoticons.
**GI** General Inquirer [93] attaches syntactic, semantic, and pragmatic information to part-of-speech tagged words. It contains 11, 788 words collected from the Harvard IV-4 dictionary and Lasswell value dictionary, which are assigned to 182 specified categories.
**GALC** Geneva Affect Label Coder [94] recognizes 36 emotion categories of affective states commonly distinguished by 267 word stems. The Geneva Emotion Wheel model [7, 8] is followed, and the 20 of the GALC categories plus an additional dimension for non-emotional words are adopted.
**OL** The Opinion Lexicon [95] is widely used by researchers for opinion mining. It consists of 2, 006 positive and 4, 783 negative words, along with the misspellings, morphological variants, slang, and social media markups.
**SWN** SentiWordNet [96] is a lexical resource for sentiment and opinion mining. It assigns to each synset of WordNet [97] three sentiment scores: positivity, negativity, and objectivity, in terms of probability.
**SS** SentiStrength [98] is a tool for automatic sentiment analysis on short social web texts written in informal language, incorporating intensity dictionaries, words with non-standard spellings, emoticons, slang and idioms.
**VADER** As a novelty, the study also adopts the Valence Aware Dictionary and sEntiment Reasoner [99]. VADER is a lexicon specifically attuned for social media texts. It has 3, 345 positive and 4, 172 negative terms, and is enhanced with general heuristics for capturing sentiment intensity.

Sentiment features are built as follows. For each categorical lexicon, a sentiment feature is represented by the histogram of all its predefined categories. Take LIWC as an instance, the generated feature vector of 93 dimensions contains numeric statistics of a review corresponding to each predefined category. Similarly, a feature vector derived from GI and GALC contains 182 and 21 elements encoding information of a review towards individual predefined categories, respectively.

As for valence-based lexicons, a review is described using a three-dimensional vector: the percentage of positive, neutral, and negative sentences in a review. Given a sentence, all its words are looked up in a lexicon, and the corresponding valence values are subsequently summed up. A sentence is considered positive if the total valence is greater than zero, negative if less than zero, and neutral otherwise. During the valence lookup, VADER heuristics are applied to OL and SWN to improve the detection accuracy [100]. The heuristics does not apply to SS since the toolkit offers a similar built-in mechanism for sentiment intensity evaluation.

The aforementioned sentiment features differ one another. In category-based lexicons, the sentiment of a review is described using predefined categories, similar to an opinion is understood from different perspectives. Meanwhile, valence-based lexicons detect the polarity of review words differently. For example, the term “clean” can be positive in some lexicons but neutral in others. As a result, the same review will obtain different vector representations due to various sentiment measurement criteria. Further details of the lexicon composition, such as the predefined categories and vocabulary can be found in the corresponding literature of individual lexicon and the survey papers [100, 101].

#### Readability

Readability measures the ease of reading texts. As pointed out by [102], even a minor increase in readability largely improves review readership, leading to more opportunities for reviews to receive helpful votes. Thus, readability has been frequently addressed in the past papers on helpfulness prediction. The following six formulas are used to construct the readability features, taking advantage of the number of characters, syllables, words, complex words, and sentences.

$$\text{FKRE}\;\left[103\right]=206.835-1.015(\frac{\#\text{words}}{\#\text{sentences}})-84.6(\frac{\#\text{syllables}}{\#\text{words}})-15.59,$$ (1)

$$\text{FKGL}\;\left[104\right]=0.39(\frac{\#\text{words}}{\#\text{sentences}})+11.8(\frac{\#\text{syllables}}{\#\text{words}}),$$ (2)

$$\text{GFI}\;\left[105\right]=0.4[(\frac{\#\text{words}}{\#\text{sentences}})+100(\frac{\#\text{complex}\;\text{words}}{\#\text{words}})],$$ (3)

$$\text{SMOG}\;\left[106\right]=1.0430\sqrt{\#\text{complex}\;\text{words}\times \frac{30}{\#\text{sentences}}}+3.1291,$$ (4)

$$\text{ARI}[107]=4.71(\frac{\#\text{characters}}{\#\text{words}})+0.5(\frac{\#\text{words}}{\#\text{sentences}})-21.43,$$ (5)

CLI[108]=0.0588L-0.296S-15.8, (6)

where complex words have at least three syllables, $L=\frac{\#\text{characters}}{\#\text{words}}\times 100$, $S=\frac{\#\text{sentences}}{\#\text{words}}\times 100$. The *z*-score is calculated for each feature for normalization.

Similar to the sentiment category, the six readability features used in the study will obtain different vector representations. While referring to the same underlying concept (ease of readiness), the use of different formulas, namely linear transformations of the counting statistics, reflects different focuses on understanding the readability of a review. Interested readers can access detailed information regarding readability tests in [109].

#### Structure

Structural features count the length and occurrence of specific language unit types. The following six features are selected to represent the structure of a review. The first three features are self-explanatory, including the number of characters (CHAR), tokens (WORD), and sentences (SENT). Similarly to Xiong et al. [110], the percentage of exclamatory (EXCLAM) and interrogative (INTERRO) sentences is taken into account. Finally, the number of misspelling words (MIS) in a review is considered.

#### Syntax

Syntactic features consider specific types and patterns of parts-of-speech within the review content. The percentage of the most prevalent open-class word categories, namely nouns (NOUN), adjectives (ADJ), verbs (VERB), and adverbs (ADV) is estimated. Additionally, the percentage of comparative sentences (COMP) is calculated. The procedure for comparative sentence detection follows the work by Jindal et al. [111], which employs a list of keywords and patterns to match the review sentences. Given that comparisons can take place implicitly, only explicit expressions are captured.

### Feature selection for helpfulness prediction

Feature-based helpfulness prediction is formulated as a binary classification (either helpful or unhelpful) problem. Most existing studies approach the task either by classification or regression. This study adopts the former due to its intuitive and simple output to customers.

The task of feature-based helpfulness prediction is formally defined as follows. Let $D=\{\left(D_{1},u_{1}\right),\ldots ,\left(D_{n},u_{n}\right)\}$ be a collection of *n* product reviews, where *D* is the content of a review and *u* the accompanying helpfulness information (*u* = 1 helpful and *u* = 0 unhelpful). Each review content D∈D is associated with a set of features, denoted by $F\left(D\right)=\{f_{1}\left(D\right),\ldots ,f_{m}\left(D\right)\}$, via *m* different feature extractors {*f*}. The goal of the task is to train a binary classifier *C* that searches for the optimal feature combination $\hat{F}$ from the feature pool F to approximate the helpfulness *u* such that:

$$\begin{array}{c} \hat{F}=\underset{F^{\prime }\subseteq F\left(D\right)}{\text{arg}\;\text{max}}\sum_{D\in D}1(u=C(F^{\prime })), \end{array}$$ (7)

where 1(·) is an indicator function. Ideally, the search of $\hat{F}$ would exhaust all possible feature combinations. Though, such a scenario is not suitable for *m* = 30 features due to the exponential complexity of calculation.

Instead, the search is fulfilled by a wrapper method, specifically, the step forward feature selection. Given the feature pool, the search starts with the evaluation of each feature and selects the one with the highest performance. Subsequently, all possible combinations between the selected feature and each of the remaining features are evaluated, and the second feature is selected. The iteration continues until adding features cannot improve the prediction performance. As a result, the selected features together form the optimal feature combination.

As for the classifier *C*, the linear Support Vector Machine (SVM) algorithm is chosen given its wide adoption and high performance in previous studies on the task [5, 9, 23, 84]. Using the most common linear SVM classifier also facilitates fair comparison between the studies within the same field.

## Empirical analysis

This section conducts substantial helpfulness prediction analysis using the 30 identified content-based features. The large-scale publicly available datasets and implementation details is introduced. The empirical results are discussed and further summarized to obtain insights into feature selection for helpfulness prediction.

### Datasets

The analysis is conducted on the largest publicly available Amazon 5-core dataset [112]. Amazon is the largest Internet retailer, which has accumulated large-scale user-generated reviews. The helpfulness of such reviews is rated by online customers, which makes it an ideal candidate for review helpfulness prediction task. In fact, Amazon product reviews are predominantly used and analyzed in previous studies. Thus, adopting Amazon reviews allows for fair comparisons with previous studies.

The original dataset consists of 24 domains, covering 142.8 million reviews collected between May 1996 and July 2014. The six domains with the highest number of reviews are selected for the study. Table 2 presents the *helpful* versus *unhelpful* review examples, where each review contains (i) a summary headline, (ii) a review text commenting in detail on the product, and (iii) the helpfulness information, namely the number of helpful and unhelpful votes given by online customers. During analysis, content-based features are extracted from the combination of the summary headline and review text.

**Table 2:** Example helpful and unhelpful reviews.

Helpful	Unhelpful
actually free!!!	are you serious?!!?
i thought for sure after downloading this app that i would have to creat an account and give away unnessecary information but surprisingly the only thing that was required was an email address and a password and i was instantly watching movies also from other reviews i was worried this app wouldnt have much to choose from but was again delightfully surprised they had more than a few good movie titles	I love hangman personally. when I saw this app I was like hmmm… and then NINETYNINE DOLLARS ARE YOU KIDDING ME? I don’t care how ultimate this is. no app is worth 99$ especially hangman! I mean the graphics are cool! I could see like 2.99 at the most but I would never buy any app for 100 dollars. really? I thought you guys were really stupid but this is ridiculous! God I cant believe it! no no no no.
825 of 906 people think this review is helpful.	3 of 131 people think this review is helpful.

Typos in the reviews are intentionally preserved.

The following pre-processing steps are performed: (1) All blank and non-English reviews are filtered out; and (2) regarding identical and nearly identical reviews [113] common on Amazon, only the ones with the highest number of votes are retained (two reviews are nearly identical if more than 80 percent of their bigram occurrence is shared [5]); and (3) the reviews with less than 10 votes are skipped to alleviate the effect of words of few mouths [114]; and (4) the remaining reviews are lowercased, tokenized, and the articles are removed.

The helpfulness label of the pre-processed reviews is determined via human assessment. For each domain, a review is labeled as unhelpful if its ratio of helpful votes is fewer than a pre-defined threshold and otherwise helpful. The threshold is set to 0.6, which is the most commonly used threshold in prior research [9, 10, 65]. To avoid the class imbalance problem, which is outside the scope of this study, the same number of helpful and unhelpful reviews are sampled.

Finally, reviews in each domain are partitioned using random stratified split: 80%, 10%, and 10% of the reviews are randomly (with a fixed seed) selected respectively for training, validation, and testing, while preserving the percentage of samples for each class. During analysis, all feature combinations are trained on the training set, compared and selected on the validation set, and evaluated on the test set serving as unseen data in reality.

**Fig 1:**
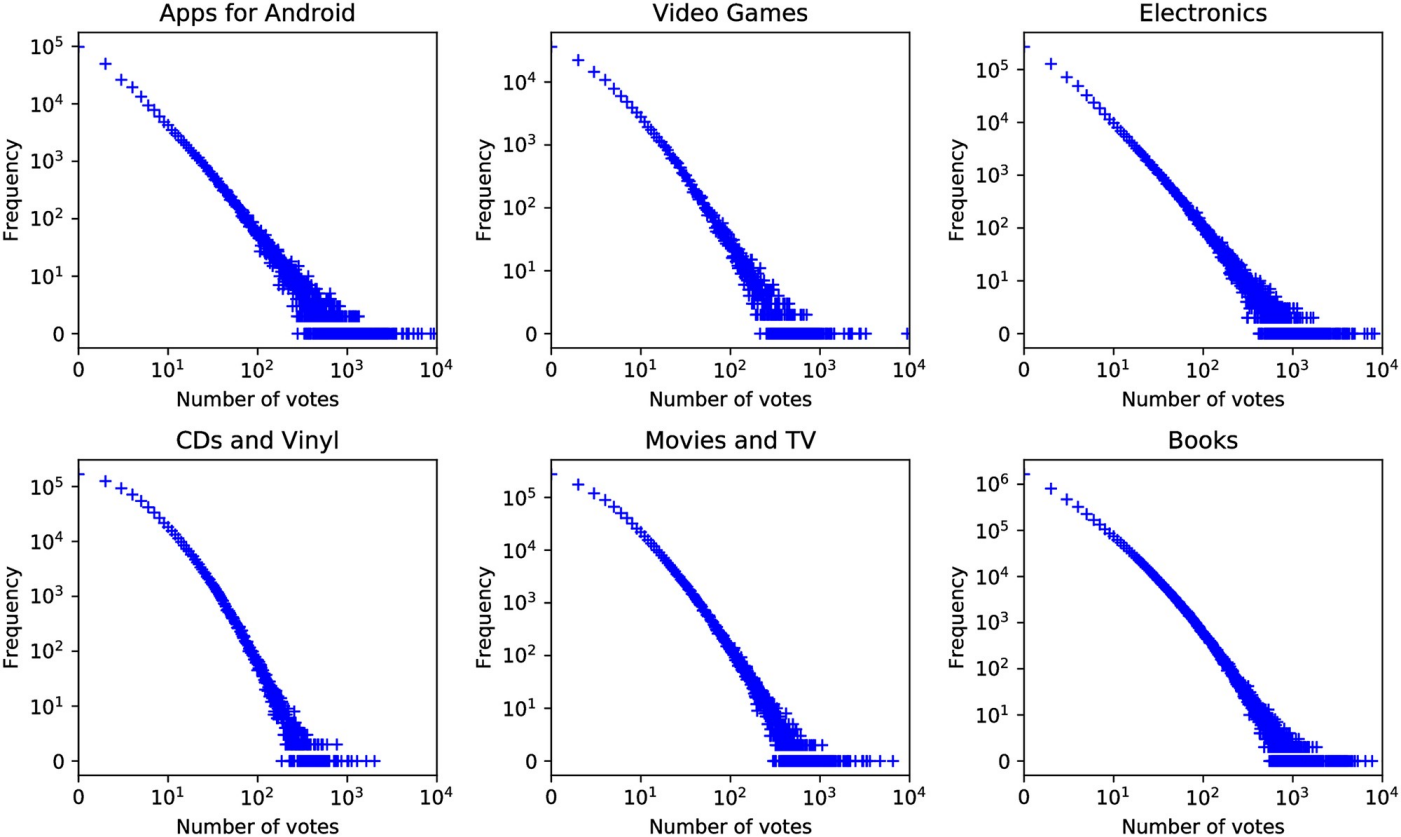
Vote distributions.

Table 3 demonstrates the descriptive statistics and out-of-vocabulary (OOV) rate of the six domains sorted by data size in ascending order. The vote distributions are further presented in Fig 1, displaying a similar pattern for each domain that high frequency of reviews have a relatively low number of votes.

**Table 3:** Descriptive statistics of the balanced datasets after pre-processing.

	Domain	#Reviews	#Tokens	#Sentences	$\frac{\#\text{Tokens}}{\#\text{Reviews}}$	$\frac{\#\text{Tokens}}{\#\text{Sentences}}$	$\frac{\#\text{Sentences}}{\#\text{Reviews}}$	OOV Rate
D1	Apps for Android	20,416	1,184,650	107,702	58.03	11.00	5.28	1.68% / 1.74%
D2	Video Games	23,100	7,522,835	469,856	325.66	16.01	20.34	0.86% / 0.86%
D3	Electronics	33,962	8,255,411	537,996	243.08	15.34	15.84	0.84% / 0.91%
D4	CDs and Vinyl	105,934	23,096,933	1,468,718	218.03	15.73	13.86	0.77% / 0.76%
D5	Movies and TV	164,052	40,549,434	2,510,899	247.17	16.15	15.31	0.62% / 0.64%
D6	Books	306,430	71,632,822	4,405,047	233.77	16.26	14.38	0.51% / 0.51%

OOV rate indicates the ratio of terms in the vocabulary of the training set is missing from the validation/test set.

### Implementation

All analysis tasks are implemented with Python 3.6 and run on Ubuntu 16.04. Text pre-processing, part-of-speech tagging, and feature extraction are done using NLTK [115]. Specifically, both SGNS trained on 100 billion words from Google News and GV trained on 840 billion words from Common Crawl are publicly available online. Regarding the sentiment category, LIWC 2015, the commercial version (February 2017) of SentiStrength, and VADER 3.2.1 are employed. The remaining lexicons are acquired as per the papers. All the readability scores are computed via the textstat library. The Hunspell spell checker is used to detect misspelling words. To enable the detection for product brands and contemporary language expressions, Hunspell is extended with Wikipedia titles (Retrieved February 13, 2019, from Wikimedia dump service). The linear SVM classifier [116] is developed using Scikit-learn [117]. For reproducibility, all randomization processes involved in the study are initialized with the same random seed.

### Results and discussion

The study considers three scenarios for feature selection: (i) individual features, (ii) features within each category, and (iii) all features. The research questions investigated can be formulated as follows:

***RQ1***: *What is the effect of individual features on review helpfulness prediction across domains?*
***RQ2***: *What are the optimal combinations of features within a category for review helpfulness prediction across domains?*
***RQ3***: *What are the optimal combinations of all features for review helpfulness prediction across domains?*
***RQ4***: *Are there any patterns of features/feature combinations for review helpfulness prediction that perform well in general?*

RQ1, RQ2, and RQ3 are answered one in a subsection. As for RQ4, the combination patterns and selection guidelines (if any) are discussed at the end of each subsection.

Throughout the analysis, the performance of review helpfulness prediction is measured by classification accuracy and its ranking. The latter is provided as another prioritization measure to capture the general trend of feature performance since the accuracy of a feature (set) can largely vary in domain.

#### RQ1: The predictive power of individual features

Table 4 demonstrates the classification accuracy, in-category ranking, and overall ranking of individual features, respectively. As shown, the semantics and sentiment category in general perform better than the other three categories.

**Table 4:** The classification accuracy and ranking (In-category/Overall) of individual features.

Category	Feature	D1		D2		D3		D4		D5		D6	
		Acc.	Rank.	Acc.	Rank.	Acc.	Rank.	Acc.	Rank.	Acc.	Rank.	Acc.	Rank.
Semantics	UGR	66.06	3 / 4	74.98	2 / 2	74.1	1 / 1	80.83	3 / 3	78.06	1 / 1	75.02	1 / 1
	BGR	60.72	4 / 10	70.74	4 / 5	69.07	4 / 5	76.89	4 / 5	74.37	4 / 4	71.03	4 / 4
	LDA	55.39	5 / 12	67.23	5 / 7	63.1	5 / 10	65.49	5 / 11	63.39	5 / 10	61.67	5 / 9
	SGNS	67.58	2 / 2	75.15	1 / 1	73.28	3 / 3	81.02	2 / 2	77.26	3 / 3	73.91	3 / 3
	GV	68.66	1 / 1	74.94	3 / 3	73.34	2 / 2	81.06	1 / 1	77.41	2 / 2	74.04	2 / 2
Sentiment	LIWC	66.16	1 / 3	73.94	1 / 4	70.78	1 / 4	76.99	1 / 4	72.25	1 / 5	68.58	1 / 5
	GI	63.76	2 / 5	69.18	2 / 6	67.07	2 / 6	72.07	2 / 6	70.75	2 / 6	67.04	2 / 6
	GALC	55.88	7 / 11	53.81	7 / 28	58.21	7 / 21	56.14	7 / 27	54.86	7 / 27	55.17	7 / 23
	OL	62.78	4 / 7	62.99	4 / 17	66.83	3 / 7	70.13	3 / 7	66.57	3 / 7	63.64	3 / 7
	SWN	61.41	5 / 8	63.12	3 / 16	62.54	5 / 12	65.29	6 / 13	63.87	5 / 9	60.92	5 / 10
	SS	60.77	6 / 9	58.05	6 / 22	60.86	6 / 19	65.6	5 / 10	61.23	6 / 17	60.6	6 / 11
	VADER	63.42	3 / 6	62.55	5 / 18	64.6	4 / 8	67.68	4 / 8	65.32	4 / 8	62.51	4 / 8
Readability	FKRE	52.99	4 / 23	58.44	3 / 21	55.36	5 / 26	62.38	4 / 20	59.15	5 / 24	53.64	6 / 27
	FKGL	51.22	6 / 28	56.84	5 / 25	55.71	4 / 25	62.12	5 / 22	59.25	4 / 23	54.92	4 / 25
	GFI	53.48	2 / 21	56.15	6 / 26	53.18	6 / 29	61.66	6 / 23	58.48	6 / 26	54.16	5 / 26
	SMOG	53.23	3 / 22	61.34	2 / 20	59.12	1 / 20	64.8	1 / 15	61.32	1 / 16	56.45	1 / 21
	ARI	51.37	5 / 27	57.1	4 / 24	55.83	3 / 24	63.15	3 / 19	59.46	3 / 22	55.57	2 / 22
	CLI	53.82	1 / 19	62.08	1 / 19	56.45	2 / 22	64.58	2 / 16	60.72	2 / 19	54.98	3 / 24
Structure	CHAR	54.36	3 / 16	65.24	1 / 9	62.07	1 / 13	64.85	1 / 14	62.56	1 / 13	58.79	2 / 14
	WORD	54.55	2 / 15	64.76	2 / 11	61.86	2 / 14	63.95	2 / 17	62.17	2 / 14	58.87	1 / 13
	SENT	52.69	5 / 26	63.94	3 / 15	61.8	3 / 15	61.64	3 / 24	60.51	3 / 20	57.94	3 / 18
	AVG	52.94	4 / 24	57.71	4 / 23	56.18	4 / 23	59.83	4 / 26	58.87	4 / 25	56.59	4 / 20
	EXCLAM	51.13	6 / 29	52.81	7 / 30	53.86	6 / 28	53.99	6 / 29	52.79	6 / 29	53.05	5 / 28
	INTERRO	55.29	1 / 13	53.16	6 / 29	51.32	7 / 30	53.43	7 / 30	53.24	5 / 28	51.8	6 / 29
	MIS	50.78	7 / 30	54.29	5 / 27	55.27	5 / 27	55.72	5 / 28	52.35	7 / 30	50.51	7 / 30
Syntax	NOUN	54.06	2 / 17	64.94	2 / 10	61.77	3 / 16	65.38	2 / 12	63	2 / 12	58.92	1 / 12
	VERB	53.72	4 / 20	64.2	4 / 13	61.09	5 / 18	62.27	4 / 21	60.81	4 / 18	58.41	4 / 17
	ADJ	55.14	1 / 14	65.41	1 / 8	63.27	1 / 9	65.63	1 / 9	63.11	1 / 11	58.65	2 / 15
	ADV	52.89	5 / 25	64.16	5 / 14	61.74	4 / 17	61.04	5 / 25	60.24	5 / 21	57.76	5 / 19
	COMP	53.97	3 / 18	64.33	3 / 12	62.89	2 / 11	63.6	3 / 18	61.56	3 / 15	58.56	3 / 16

**Semantics** The semantics category consists of most of the globally top-five features. The best overall performance lies in semantic features directly modeling review content, leading to more dimensions for encoding information. In particular, UGR sets a strong baseline in all domains, which indicates that specific term occurrences differ between helpful and unhelpful reviews. Both GV and SGNS show comparable or higher performance than UGR, with about 1% in accuracy lower than UGR in the worst case. The promising performance demonstrates the efficacy of traditional machine learning algorithms trained on general-purpose distributed word representations for helpfulness prediction. GV outperforms SGNS in all domains except D2, being a preferable option. In contrast, BGR scores 4%–5% lower compared with UGR, suggesting increased data sparsity while using bigram features. LDA consistently ranks the lowest within the category and is even lower than several features in the sentiment and syntax category. The inferior performance can be attributed to short product reviews hindering the training of topic distributions, which explains the lowest (highest) overall LDA ranking on D1 (D2).
**Sentiment** The sentiment category shows mixed performance. As for the categorical lexicons, LIWC, GI, and GALC rank respectively first, second, and last in all domains. LIWC outperforms UGR in D1 but is beaten by other domains. The accuracy gap, ranging from 1% to 6%, is proportional to data size. As such, LIWC can substitute for semantics when applied to small datasets. While the drastic low performance of GALC results from its few predefined categories and low vocabulary coverage compared with LIWC, GI shows that having almost double the size of predefined lexicon categories and words does not necessarily bring higher performance. On the other hand, the valence-based lexicons perform variously depending on data size. In most cases, OL and VADER produce higher accuracy than SWN and SS. Starting from D3, a more precise pattern that OL>VADER>SWN>SS is observed. OL generally performs better than other valence-based lexicons because it is originally generated from Amazon reviews, and thus more related to the tested domains. The results from the category show that the predictive power of lexicon-based features highly depends on the definition of lexicon categories, vocabulary coverage, as well as data size.
**Readability, Structure and Syntax** Features from the remaining three categories generally have less individual predictive power. The majority of the features have lower rankings, with the accuracy about 10%-27% inferior to UGR. The low performance indicates the indistinguishable nature among classes. In the readability category, for instance, similar scores are observed regardless of helpfulness of a review. Likewise, both helpful and unhelpful reviews are characterized by similar ratio of exclamatory and interrogative sentences, as well as misspellings. As a result, such features are less preferable in the helpfulness prediction task when used individually. Still, the slightly improved accuracy in the syntax category indicates that helpfulness is more related to the proportion of open-class words. In particular, ADJ generally performs better than other syntactic features due to the descriptive nature of products or general purchase satisfaction/dissatisfaction.

**Fig 2:**
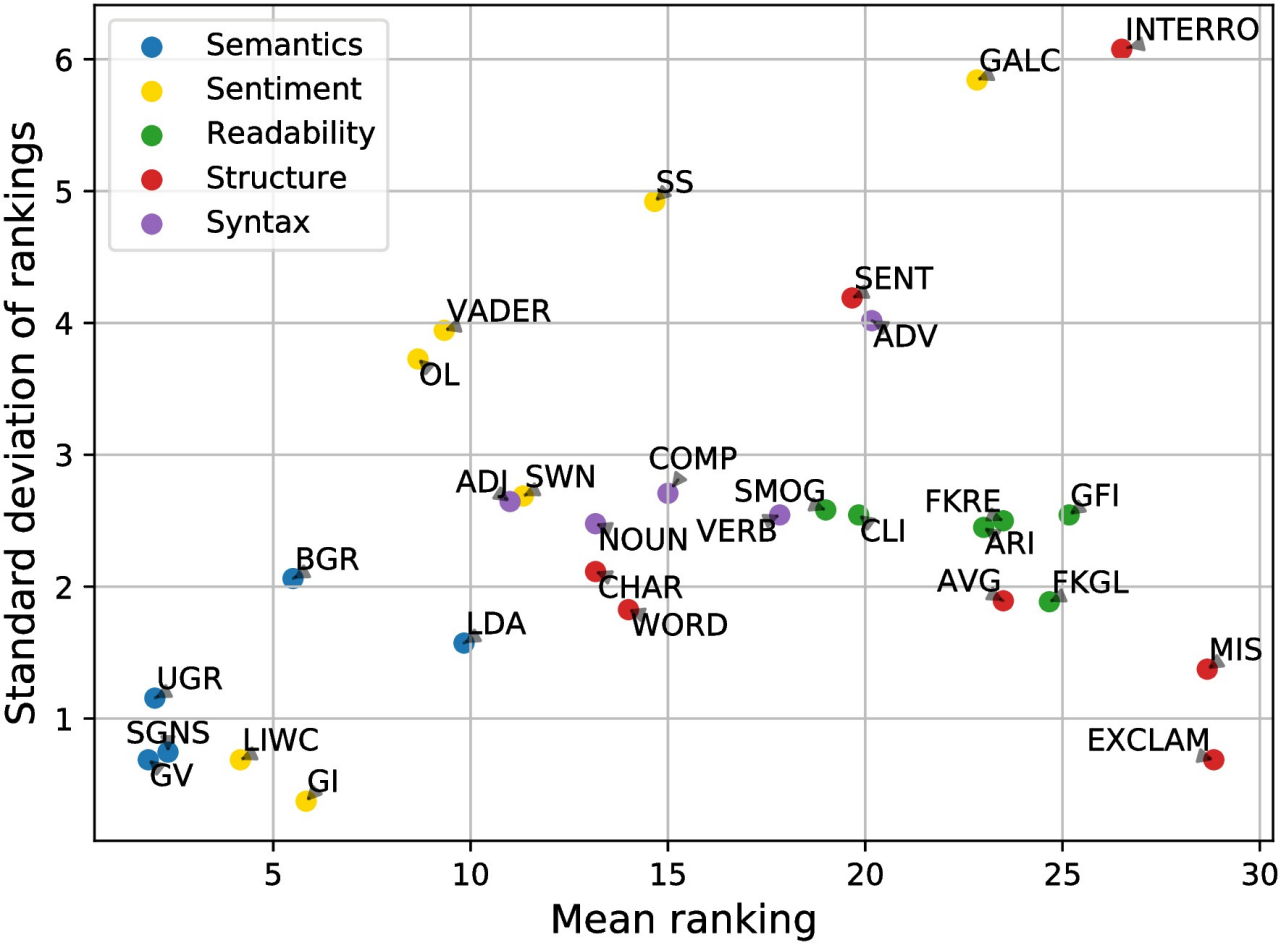
Feature performance versus stability.

To better understand the behaviour of individual features across domains, the mean and standard deviation of the overall ranking of each feature are produced in Fig 2. The former describes the average performance of a feature, whereas the latter describes the stability of feature performance. As demonstrated, GV, SGNS, UGR, LIWC, and GI are the most ideal features with both excellent performance and stability. Those features show the feasibility of helpfulness prediction by modeling semantics and sentiment of product reviews. The remaining features, however, have either less satisfactory or stable performance.

**Summary** The findings and guidelines for review helpfulness prediction using individual features are summarized below:

1. Consider UGR, GV, and SGNS in the semantics category with higher priority since they are the most distinctive for informative reviews. In particular, GV performs better than SGNS in most cases.
2. Features in the sentiment category are less effective in review helpfulness prediction compared with the three semantic counterparts. However, it is worth trying to replace the semantics with LIWC in small datasets.
3. Most features in the structure, readability, and syntax category are of minor predictive power and not suggested to use individually.

#### RQ2: Optimal combinations of features within each category

Table 5 presents the accuracy and ranking of the optimal feature combination in each category. As shown, BGR is the only feature not being selected in any scenarios due to the associated sparsity. Also, all domains demonstrate an identical ranking of feature categories, with the semantics, sentiment, and structure category playing the dominant role in helpfulness prediction.

**Table 5:** Optimal combinations of features within each category.

	Category	Accuracy	Ranking	Combination
D1	Semantics	67.53	1	GV+SGNS
	Sentiment	67.29	2	LIWC+OL+GI
	Readability	54.06	5	SMOG+CLI+FKGL
	Structure	56.51	3	SENT+INTERRO+EXCLAM+AVG+WORD+CHAR
	Syntax	55.14	4	ADJ+ADV+COMP
D2	Semantics	76.67	1	SGNS+GV+LDA
	Sentiment	74.81	2	LIWC+VADER
	Readability	64.46	5	CLI+SMOG+GFI+ARI+FKGL
	Structure	66.67	3	CHAR+WORD+MIS+INTERRO
	Syntax	65.54	4	NOUN+ADJ+ADV+VERB
D3	Semantics	74.10	1	GV+SGNS
	Sentiment	73.98	2	LIWC+OL+GI+GALC+VADER
	Readability	59.12	5	SMOG
	Structure	64.10	3	CHAR+INTERRO+WORD
	Syntax	63.60	4	ADJ+ADV
D4	Semantics	81.32	1	UGR+GV
	Sentiment	78.81	2	LIWC+OL+GI+SS+SWN+VADER
	Readability	65.52	5	SMOG+ARI+FKGL
	Structure	68.68	3	CHAR+INTERRO+WORD+MIS+SENT
	Syntax	67.36	4	ADJ+ADV+VERB+NOUN
D5	Semantics	78.18	1	UGR+GV
	Sentiment	74.95	2	LIWC+GI+OL+VADER+SS+SWN
	Readability	62.05	5	SMOG+CLI+GFI+ARI+FKRE
	Structure	65.92	3	CHAR+INTERRO+WORD+EXCLAM+MIS
	Syntax	63.89	4	NOUN+VERB+ADV+COMP
D6	Semantics	75.49	1	UGR+SGNS+LDA
	Sentiment	71.45	2	LIWC+GI+OL+SWN+SS+GALC
	Readability	59.05	5	SMOG+ARI+FKRE+CLI+FKGL
	Structure	61.59	3	WORD+INTERRO+MIS+EXCLAM+SENT
	Syntax	59.11	4	NOUN+COMP+VERB+ADV

**Fig 3:**
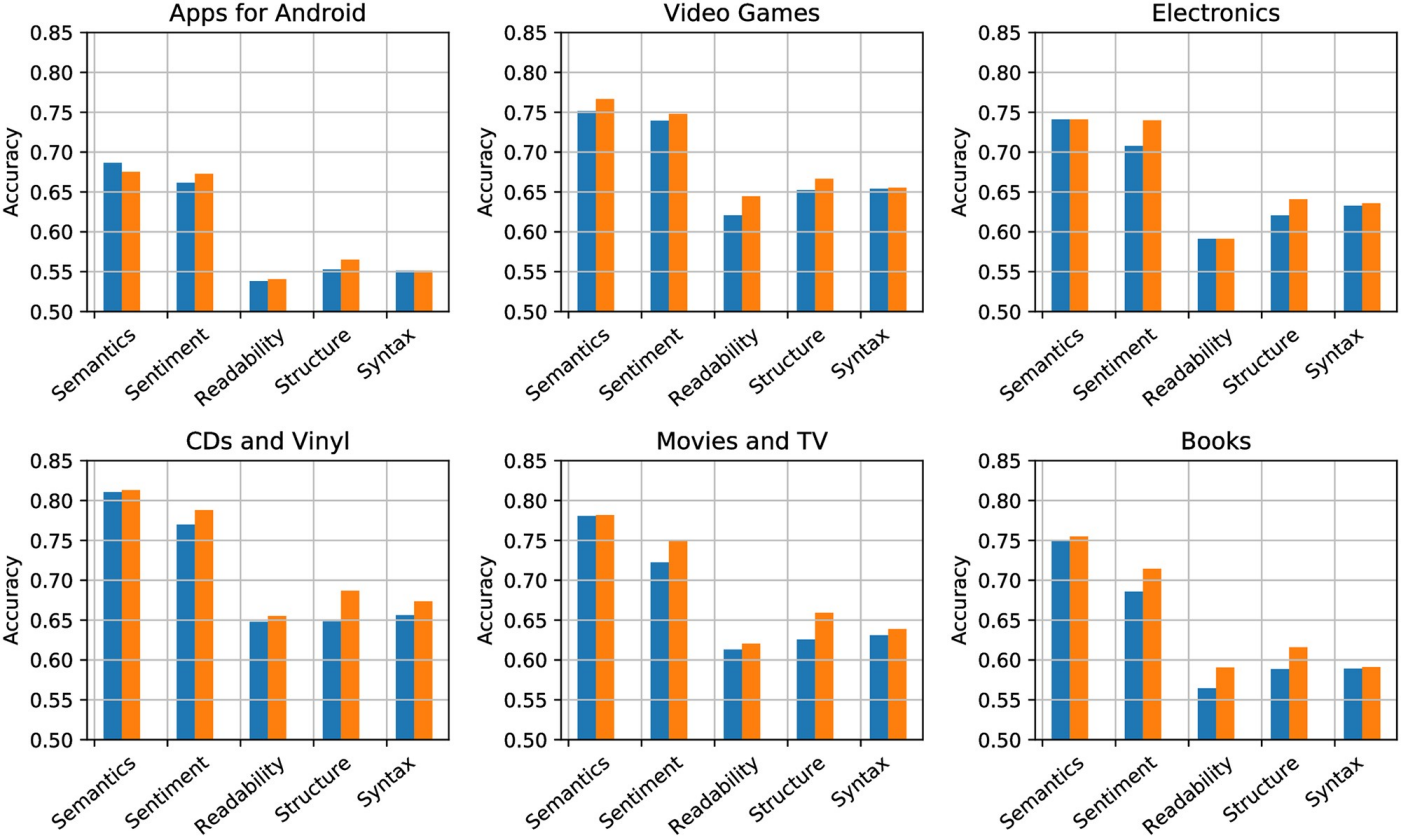
Comparison between max-performance individual feature (Blue) and max-performance combination of features (Orange) within each category.

To evaluate the benefit of combining multiple features within the same category, the optimal feature combination is compared with the most promising individual feature. As Fig 3 illustrates, in all but one cases, using multiple features achieves better performance on a category level. The rationale is that combining features provides new descriptive information of reviews and allows the information to complement one another. The improvement, depending on domains, tends to be more noticeable in the sentiment, readability, and structure category. On D1, GV alone reports higher accuracy than the optimal combination GV+SGNS in the semantics category since the domain has a large proportion of OOV words. As shown in Table 3, D1 has the shortest average length but highest OOV rate, which is about twice as much as other domains. Further manual inspection reveals that many OOV words are domain-specific terms such as names of mobile applications and mobile games. Moreover, only 53% of the OOV words overlap between the validation and test set. When converting reviews into embeddings, the OOV issue in the pre-trained SGNS model further affects the performance, which explains why GV+SGNS is worse than GV and less robust on D1.

The average number of features within each category used for helpfulness prediction is provided in Table 6. Frequent feature combination patterns that occur at least four times across domains are extracted via the PrefixSpan algorithm. The constant use of LIWC, SMOG, ADV, and INTERRO+WORD is observed, and thus it is recommended to include them for optimal feature combinations within the corresponding categories. As for the sentiment category, adding GI+OL (VADER alone) on top of LIWC can achieve higher performance in five (four) of six domains. Similarly, using INTERRO+WORD in conjunction with CHAR (MIS) can improve the structure category in five (four) domains. Furthermore, including one of ARI, CLI, and FKGL in addition to SMOG in the readability category helps to increase the accuracy in four domains. The same applies to ADJ and NOUN+VERB for ADV in syntax category. Finally, the semantics category tends to have various feature combinations, with GV and SGNS prevalent in most cases.

**Table 6:** Frequent feature combination patterns within each category.

Category	#Features	Pattern (Frequency)
Semantics	2.33 ± 0.47	GV (5);
Sentiment	4.67 ± 1.60	LIWC (6); GI+OL+LIWC (5); LIWC+VADER (4)
Readability	3.67 ± 1.49	SMOG (6); ARI+SMOG (4); CLI+SMOG (4); FKGL+SMOG (4)
Structure	4.67 ± 0.94	INTERRO+WORD (6); CHAR+INTERRO+WORD (5); INTERRO+MIS+WORD (4)
Syntax	3.50 ± 0.76	ADV (6); ADJ+ADV (4); ADV+NOUN+VERB (4)

**Summary** The findings and guidelines for review helpfulness prediction using multiple features within each category are summarized below:

1. The optimal combination of semantic features consistently outperforms those in other categories in helpfulness prediction. Specifically, it is suggested that the combination includes GV as the first feature.
2. Regarding the sentiment (structure) category, it is recommended the optimal combination base on LIWC (INTERRO+WORD), and subsequently follow an addition of OL+GI (CHAR alone) to the corresponding category since performance gains are reported in most cases.
3. In regard to the readability (syntax) category, it is suggested the optimal feature combination base on SMOG (ADV), and subsequently follow an addition of one of ARI, CLI, and FKGL (ADJ, NOUN+VERB) to the corresponding category as this generally leads to visible performance gains.

#### RQ3: Optimal combinations of all features

The final result of review helpfulness prediction using the optimal feature combination from all categories are presented in Table 7. The optimal combinations contain four to seven features selected from only 18 out of the 30 features. Some of the 12 excluded features have excellent individual performance or are popular in category-level combinations, such as GI and WORD. The exclusion is due to features selected earlier (partly) contain information provided by those later. Despite no clear-cut patterns across domains are observed from the combinations, the semantics, sentiment, and syntax category play more important role in forming the optimal feature combinations. Especially, GV, UGR, LIWC and ADJ are used on half of the occasions.

**Table 7:** Optimal combinations of all features.

	Accuracy	Sem.	Sen.	Read.	Str.	Syn.	Combination
D1	69.78	✓	✓			✓	GV+VADER+ADV+SWN
D2	76.80	✓			✓	✓	SGNS+GV+LDA+MIS+INTERRO+ADJ
D3	75.96	✓	✓	✓			GV+SGNS+LIWC+OL+GALC+ARI
D4	83.09	✓	✓	✓	✓	✓	UGR+LIWC+ARI+INTERRO+CLI+ADJ+VERB
D5	79.72	✓	✓		✓	✓	UGR+CHAR+LIWC+ADJ
D6	76.20	✓	✓	✓		✓	UGR+SMOG+ADV+VADER

**Fig 4:**
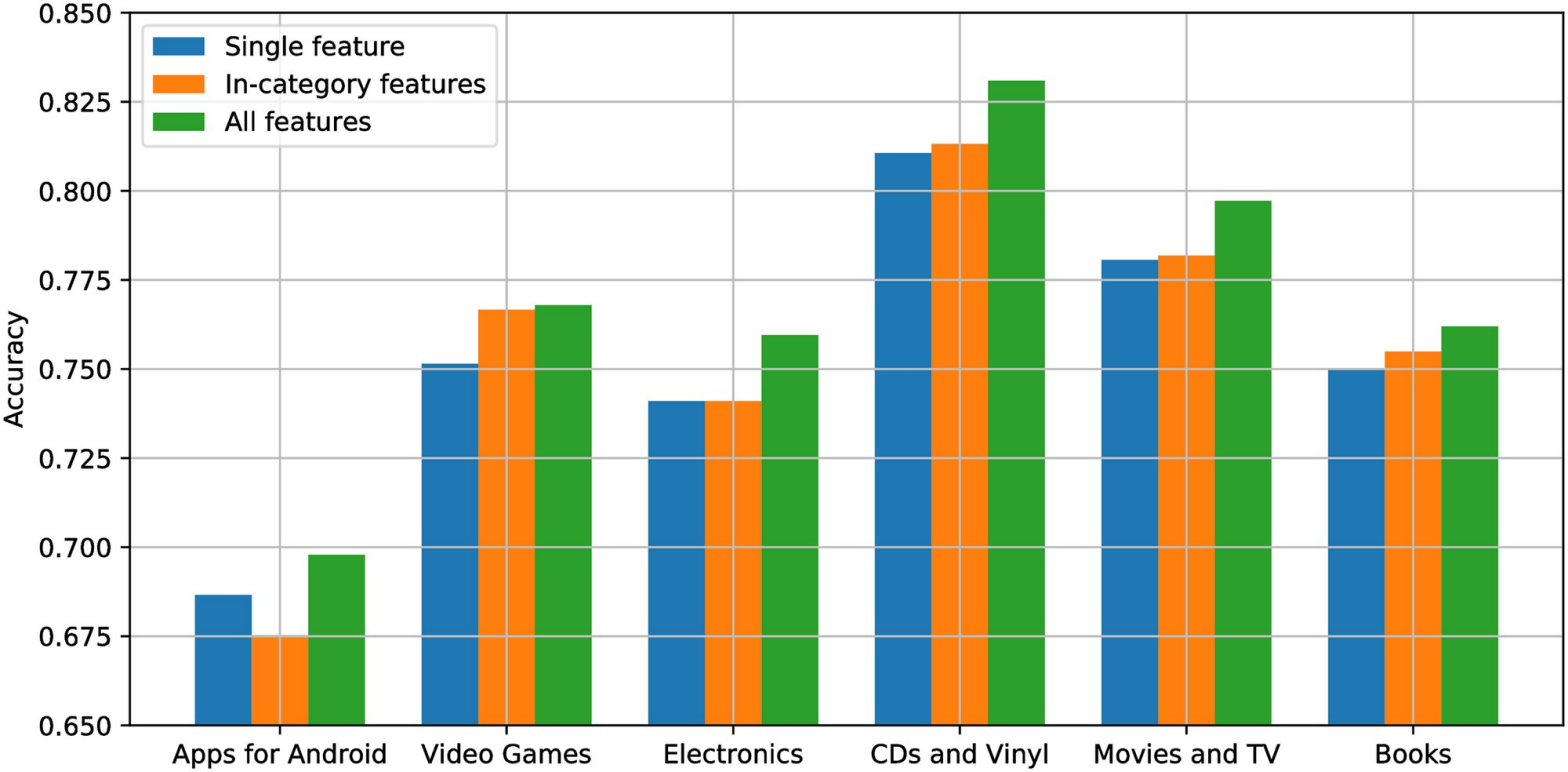
Comparison among the max-performance individual feature (Blue), max-performance in-category feature combination (Orange), and max-performance combination of all features (Green).

The accuracy among the max-performance individual feature, optimal feature combination within each category, and optimal combination of all features is further compared in Fig 4. As shown, using features from multiple categories consistently achieves the highest performance. Similar to using multiple features within a category, the improvement lies in features from different categories together describe a review from multiple perspectives, making the vector representations more comprehensive.

**Summary** The findings and guidelines for review helpfulness prediction using features from multiple categories are summarized below:

1. Initialize the optimal combination with no more than three (usually one or two) semantic features, starting with GV or UGR, followed by SGNS.
2. Extend the combination with the remaining features in a forward selection manner. It is suggested that features mentioned in Table 4 have higher priority than those that are not.
3. Finalize the search by integrating the unused features into the combination using forward selection.

## Implications

The last section has presented a series of optimal feature combinations across domains, along with their predictive power. The general guidelines for feature selection under the three scenarios are also summarized for future researchers. Extensive analysis shows that appropriately increasing the number of features can increase the performance of helpfulness prediction in almost all cases, regardless of feature categories and feature selection scenarios. As discussed, the performance gains lie in multiple features helping model a review’s helpfulness information in a more comprehensive manner.

Nevertheless, it is worth noting that the semantics category contributes largely to the final performance. Throughout the study, using UGR alone accounts for 97.96% ± 0.35% of the accuracy compared with the optimal combination of all features across domains. The exclusive use of SGNS and GV can also yield comparable prediction performance. The empirical results demonstrate that combining many of the selected features, while leading to various performance gains, does not significantly improve helpfulness prediction. This contradicts prior studies largely combining multiple features without solid and sufficient justification. The extensive feature evaluation conducted in this study fills the gap of currently arbitrary feature selection process to review helpfulness evaluation.

The success of the semantics category can be explained from two perspectives: the encoding dimensionality and encoding methods. UGR, SGNS, and GV encode review content using more dimensions than other features. For many features that only have single dimension, encoding all text information into limited vector space can be challenging. On the other hand, both SGNS and GV achieve comparable performance to UGR with far fewer dimensions, showing that the information density of a feature varies from encoding methods. Even when used jointly, features beyond the semantics category are still less representative.

The dominance of review semantics also proves the feasibility of a new helpfulness prediction direction: Instead of laborious feature engineering, potential performance gains can be hopefully achieved by modeling sole semantic features from reviews via more advanced techniques, for example, state-of-the-art deep learning algorithms.

The authors hope that the exploration of potential factors behind the helpfulness evaluation process will deepen the insights obtained and contribute toward improved prediction system development.

## Limitations

In terms of limitations, only the content-based features are considered due to their wide availability across various platforms. Also, the simplified forward selection search process for optimal feature combinations is adopted, thus not all possible scenarios are exhausted. Finally, the potential customer bias for the review helpfulness judgement (assertion of an initial belief), the common fraudulence issue (positive/negative review manipulation), as well as the sequential bias (early reviews receive disproportionately higher number of votes due to positive feedback loop [118]) are not taken into consideration due to the complex nature of such assessment.

## Conclusions and future works

With the rapid development of Web 2.0, online product reviews have become an essential source of knowledge for most customers when making e-purchase decisions. In the deluge of data, to identify and recommend the informative reviews, rather than those of random quality is an important task. Feature-based methods have long been the paradigm of helpfulness prediction due to relatively simple implementation and effective interpretability. In the study presented, the 30 most frequent content-based features from five categories have been identified, and their extensive evaluation is conducted on six top domains of the largest publicly available Amazon 5-core dataset. The individual features, feature combinations within each category, and all feature combinations that lead to optimal performance have been studied. As stated by Charrada [31], the usefulness of a review is likely to depend on numerous factors that are difficult to isolate and study. The empirical results set comparable and reproducible baselines for review helpfulness prediction, and more importantly, highlight the feature combination patterns that lead to general good prediction performance, regardless of application domain and/or source platform.

Several significant findings and guidelines in feature selection are worth highlighting. Among many features, unigram TF-IDF and the two more recent pre-trained word embeddings yield strong predictive power across all domains, demonstrating the effectiveness of encoding semantics for helpfulness prediction. The LIWC dictionary achieves the closest performance to the three semantic features with far fewer feature dimensions, showing the feasibility of helpfulness prediction with fine-grained categorical sentiments. Another important finding is that appropriately combining features from multiple categories effectively improves the performance over individual features, or features from one single category. A good rule of thumb for feature selection is to initialize the search with semantic features, followed by features mentioned in Table 4, and finally the remaining content-based features. The findings and guidelines of this work can facilitate feature selection in review helpfulness prediction.

As final contribution, the authors have open sourced the computational framework that implements a holistic solution for feature-based helpfulness prediction. The dataset split configurations, pre-processed reviews, and extracted features used in the study have also been publicly released within research community for result reproducibility, fair comparison, and future improvement. The framework can be extended to support additional methods for feature extraction, feature selection, classification, and parameter tuning, allowing for more flexible investigation on the feature behaviors. Meanwhile, the off-the-shelf extracted features can help future researchers efficiently explore many possible feature combinations for the task.

The following directions will be addressed in the future. (1) Selected context-based features and less popular content-based features that are currently excluded will be taken into account to validate their predictive power. Especially, the social connection among reviewers and reviewer characteristics (e.g., reviewer age, the number of history reviews posted by a reviewer) will be emphasized. (2) The potential extension to other domains in the 5-core Amazon dataset and other platforms such as Yelp and TripAdvisor will be included following the holistic view on helpfulness prediction task. (3) The moderating factors will be explored, such as the product type and sequential bias. As stated by Ocampo et al. [4], it is perfectly sensible to expect the helpful reviews of different product types to be different. Given the context of a review, Sipos et al. [119] found that the helpfulness votes are often the consequence of its nearest neighbours. (4) More robust and sophisticated machine learning models will be employed to select representative features for helpfulness prediction. For example, recent explainable deep learning techniques can be employed to model semantic features from review content to free helpfulness prediction studies from heavy feature engineering.
